# Multi-country evaluation of RISK6, a 6-gene blood transcriptomic signature, for tuberculosis diagnosis and treatment monitoring

**DOI:** 10.1038/s41598-021-93059-1

**Published:** 2021-07-01

**Authors:** Rim Bayaa, Mame Diarra Bousso Ndiaye, Carole Chedid, Eka Kokhreidze, Nestani Tukvadze, Sayera Banu, Mohammad Khaja Mafij Uddin, Samanta Biswas, Rumana Nasrin, Paulo Ranaivomanana, Antso Hasina Raherinandrasana, Julio Rakotonirina, Voahangy Rasolofo, Giovanni Delogu, Flavio De Maio, Delia Goletti, Hubert Endtz, Florence Ader, Monzer Hamze, Mohamad Bachar Ismail, Stéphane Pouzol, Niaina Rakotosamimanana, Jonathan Hoffmann, The HINTT working group within the GABRIEL network, Graciela Russomando, Chyntia Carolina Díaz Acosta, Rossana Arenas

**Affiliations:** 1 Medical and Scientific Department, Fondation Mérieux, Lyon, France; 2 Laboratoire Microbiologie, Santé et Environnement (LMSE), Doctoral School of Sciences and Technology, Faculty of Public Health, Lebanese University, Tripoli, Lebanon; 3 Institut Pasteur de Madagascar, Antananarivo, Madagascar; 4 Department of Biology, Ecole Normale Supérieure de Lyon, Lyon, France; 5 Equipe Pathogénèse des Légionelles, International Center for Research in Infectiology, INSERM U1111, University Lyon 1, CNRS UMR5308, École Normale Supérieure de Lyon, Lyon, France; 6 National Center for Tuberculosis and Lung Diseases (NCTLD), Tbilisi, Georgia; 7 International Centre for Diarrhoeal Disease Research, Bangladesh (icddr,b), Dhaka, Bangladesh; 8 Centre Hospitalier Universitaire de Soins et Santé Publique Analakely (CHUSSPA), Antananarivo, Madagascar; 9 Dipartimento di Scienze di Laboratorio e Infettivologiche, Fondazione Policlinico Universitario “A. Gemelli”, IRCCS, Rome, Italy; 10 Translational Research Unit, Department of Epidemiology and Preclinical Research, “L. Spallanzani” National Institute for Infectious Diseases (INMI), IRCCS, Rome, Italy; 11 Erasmus MC, Medical Microbiology and Infectious Diseases, University Medical Center Rotterdam, Rotterdam, The Netherlands; 12 Service des Maladies Infectieuses et Tropicales, Hospices Civils de Lyon, Lyon, France; 13 Instituto de Investigaciones en Ciencias de la Salud, National University of Asunción, Asunción, Paraguay; 14 Hospital General de San Lorenzo, MSPyBS, Asunción, Paraguay

**Keywords:** Infectious-disease diagnostics, Molecular biology, Diagnostic markers, Tuberculosis, Laboratory techniques and procedures

## Abstract

There is a crucial need for non-sputum-based TB tests. Here, we evaluate the performance of RISK6, a human-blood transcriptomic signature, for TB screening, triage and treatment monitoring. RISK6 performance was also compared to that of two IGRAs: one based on RD1 antigens (QuantiFERON-TB Gold Plus, QFT-P, Qiagen) and one on recombinant *M. tuberculosis* HBHA expressed in *Mycobacterium smegmatis* (IGRA-rmsHBHA). In this multicenter prospective nested case–control study conducted in Bangladesh, Georgia, Lebanon and Madagascar, adult non-immunocompromised patients with bacteriologically confirmed active pulmonary TB (ATB), latent TB infection (LTBI) and healthy donors (HD) were enrolled. ATB patients were followed-up during and after treatment. Blood RISK6 scores were assessed using quantitative real-time PCR and evaluated by area under the receiver-operating characteristic curve (ROC AUC). RISK6 performance to discriminate ATB from HD reached an AUC of 0.94 (95% CI 0.89–0.99), with 90.9% sensitivity and 87.8% specificity, thus achieving the minimal WHO target product profile for a non-sputum-based TB screening test. Besides, RISK6 yielded an AUC of 0.93 (95% CI 0.85–1) with 90.9% sensitivity and 88.5% specificity for discriminating ATB from LTBI. Moreover, RISK6 showed higher performance (AUC 0.90, 95% CI 0.85–0.94) than IGRA-rmsHBHA (AUC 0.75, 95% CI 0.69–0.82) to differentiate TB infection stages. Finally, RISK6 signature scores significantly decreased after 2 months of TB treatment and continued to decrease gradually until the end of treatment reaching scores obtained in HD. We confirmed the performance of RISK6 signature as a triage TB test and its utility for treatment monitoring.

## Introduction

One fourth of the world population is estimated to be infected with *Mycobacterium tuberculosis* (*Mtb*) that causes approximately 10 million cases of tuberculosis (TB) yearly. This disease ranks among the leading causes of death worldwide, resulting in 1.4 million deaths in 2019^1^. Five to 10% of infected individuals develop the contagious, active form of TB (ATB) disease, while most of them (90%) control the infection and develop asymptomatic latent TB infection (LTBI). However, a small proportion (10%) of LTBI individuals will develop ATB during their lifetime^2^. TB can be treated with a regimen of several antibiotics for a minimum of 6 months. In most patients, TB therapy provides cure^3^ but treatment failure and relapse can occur. These outcomes are associated with severe adverse effects and long treatment durations that induce a lack of patient adherence to the treatment regimen thus promoting the emergence of drug-resistance^4^.

Current ATB diagnostic tests include sputum-based culture and acid-fast Bacillus (AFB) smear microscopy which are also used for monitoring TB treatment response^1,3^. Molecular tests like the GeneXpert MTB/RIF or ULTRA, are also performed using sputum samples^5^. Interferon (IFN)-*γ* release assays (IGRAs) such as QuantiFERON-TB Plus (QFT-P; Qiagen) are blood-based tests used for the detection of *Mtb* infection, yet cannot discriminate ATB from LTBI^6–9^. However, the combined use of QFT-P with the heparin-binding hemagglutinin antigen; HBHA-based IGRAs, that relies on the stimulation of whole blood with recombinant *Mtb* HBHA protein expressed in *Mycobacterium smegmatis* (IGRAs-rmsHBHA)^10^, recently revealed the potential for the stratification of TB stages (e.g. ATB vs LTBI)^11–14^.

Sputum-based TB tests are associated with several limitations including the long-time of culture and the lack of sensitivity and specificity of smear microscopy^15^. Besides, although molecular tests are more sensitive for diagnosing pulmonary TB, they still have limited sensitivity in paucibacillary pulmonary TB patients^16,17^. In addition, sputum samples may be difficult to obtain in some populations (e.g. children and HIV co-infected TB patients) as well as in ATB patients after symptom improvement^18^. In this context, the World Health Organization (WHO) has declared an urgent need for alternative non-sputum-based TB tests with a series of target product profiles (TPPs) which detailed the minimal and optimal criteria that should be met to diagnose and monitor TB treatment response^19–21^. Those new TB tests need to be based on accessible biological samples such as whole blood or urine, and must be practical for field applications^22^.

Currently, there is much active research^23,24^ on human blood transcriptomic TB biomarkers^25^. A six whole blood gene transcriptomic signature (RISK6) has been recently described and validated in 7 independent cohorts, demonstrating its utility to predict the risk of progression from TB infection to ATB disease, as a screening test for TB, and to monitor TB treatment response^19,26^. The present study aims: to evaluate the robustness of the RISK6 signature in four additional independent cohorts from different countries and ethnicities; to assess its performance for TB screening and triage; to compare its performance to that of two IGRAs (QFT-P and IGRAs-rmsHBHA); and to evaluate its utility for monitoring treatment outcome.

## Results

### Sociodemographic and clinical characteristics

A total of 141 patients with bacteriologically confirmed pulmonary ATB were included in the study. Their sociodemographic and clinical characteristics were compared at baseline. The median age was 28 years, 66% were male, and 51.8% were smokers. Among them, 48.2% had a positive sputum smear microscopy with a high grade at baseline (2+ or 3+). 97 of these patients were followed at least until the end of treatment and have been successfully treated for TB. The remaining participants included 26 individuals with LTBI and 71 healthy donors (Table 1).

**Table 1:** Baseline sociodemographic and clinical characteristics of ATB patients in the four cohorts.

	Georgia	Madagascar	Lebanon	Bangladesh	Total
ATB (N)	32	44	21	44	141
**ATB patient demographics**					
Age (years)	33.5 (26.75–44.5)	29.5 (21.75–43.25)	30 (22–37)	23.5 (20.75–30.5)	28 (22–39)
Gender (male)	81.2% (26/32)	59.1% (26/44)	47.6% (10/21)	70.5% (31/44)	66% (93/141)
BMI at baseline	20.06 (18.65–21.67)	17.19 (16.31–18.67)	20.94 (19.59–21.41)	18.28 (16.2–20.79)	18.68 (16.89–20.95)
**Vaccination**					
BCG vaccination	40.6% (13/32)	88.6% (39/44)	19% (4/21)	75% (33/44)	63.1% (89/141)
**Risk factors**					
Smoking habit	59.4% (19/32)	43.2% (19/44)	57.1% (12/21)	52.3% (23/44)	51.8% (73/141)
Alcohol consumption	9.7% (3/31)	45.5% (20/44)	9.5% (2/21)	11.4% (5/44)	21.4% (30/140)
Injecting drug users	–	–	–	9.3% (4/43)	2.9% (4/138)
Jail detention history	6.2% (2/32)	2.4% (1/42)	14.3% (3/21)	4.5% (2/44)	5.8% (8/139)
**Other pathologies**					
HCV positive	9.4% (3/32)	2.3% (1/44)	–	–	2.8% (4/141)
Other underlying disease	–	9.1% (4/44)	9.5% (2/21)	2.3% (1/44)	5.5% (7/127)
**Sputum smear microscopy at baseline**					
Low grade (1+ or scanty)	37.5% (12/32)	25% (11/44)	28.6% (6/21)	27.3% (12/44)	29.1% (41/141)
High grade (2+ or 3+)	25% (8/32)	54.5% (24/44)	38.1% (8/21)	63.6% (28/44)	48.2% (68/141)
Negative	34.4% (11/32)	20.5% (9/44)	19% (4/21)	9.1% (4/44)	19.9% (28/141)
Not evaluated	3.1% (1/32)	–	14.3% (3/21)	–	2.8% (4/141)
**TB treatment**					
Treated	26	33	15	23	97
LTBI (N)	–	26	–	–	26
Healthy donors (N)	7	23	25	16	77

*TB* Tuberculosis, *BMI* Body Mass Index, *LTBI* latent TB infection, *IQR* interquartile range. Data were given as % (N) or median (IQR).

### Performance of the RISK6 signature as a screening and triage test for pulmonary TB disease

**Figure 1:**
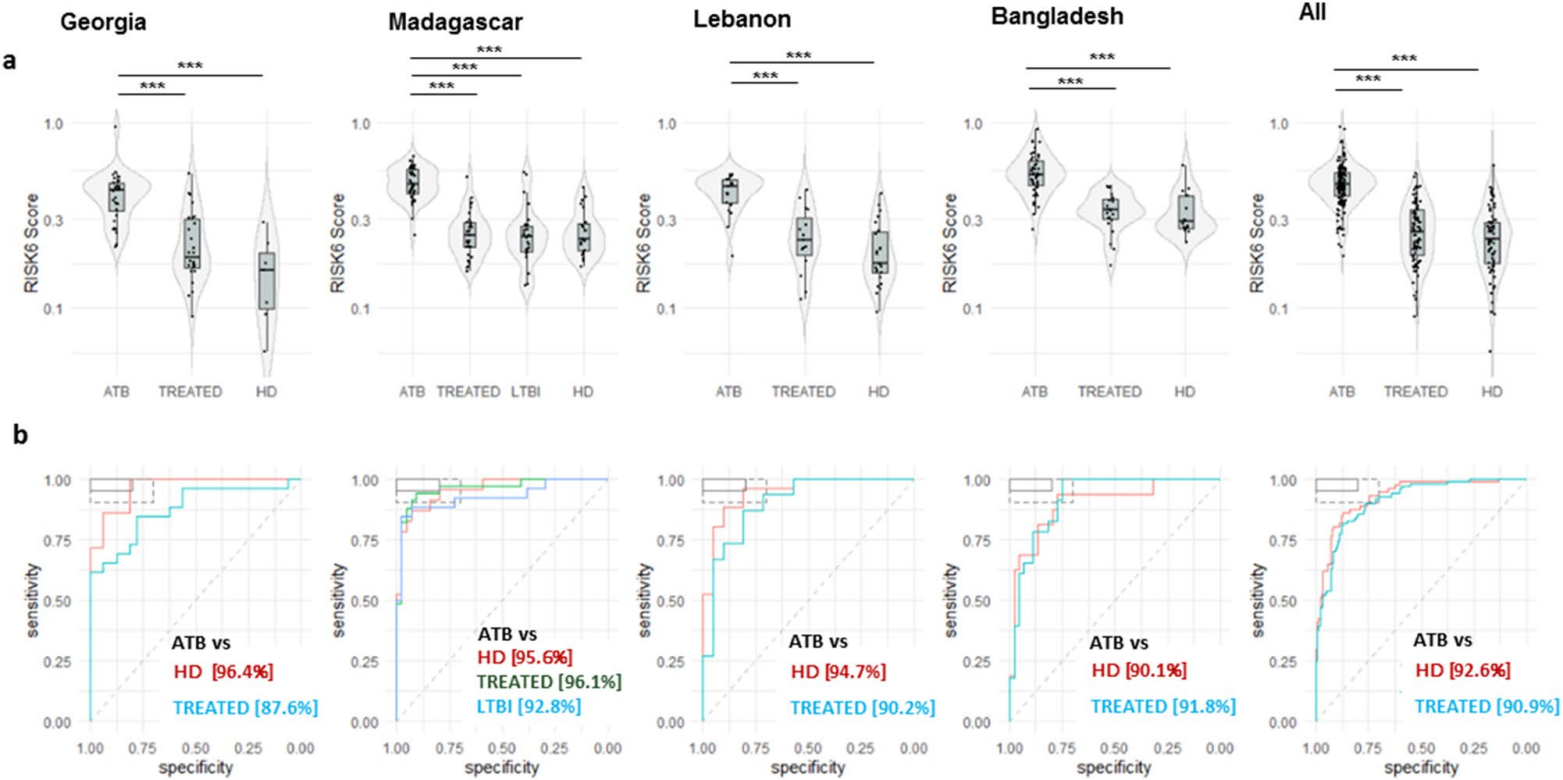
Validation of the performance of a multi-cohort 6-gene signature; RISK6 as a screening and triage test in patients with pulmonary TB. (**a**) Violin plots showing the differences in the levels of RISK6 signature scores from patients with active TB at baseline (ATB, n = 141), treated TB patients (TREATED, n = 97; patients with a negative sputum culture at T2 and/or T3), individuals with a latent TB infection (LTBI, n = 26), and healthy donors (HD, n = 71) from Georgia, Madagascar, Lebanon, Bangladesh and in all sites. Horizontal lines designate medians, boxes represent the inter-quartile ranges (IQR) and the ranges are represented by whiskers. Single patient results are represented by each dot in the graph. Statistical significance was calculated using Mann–Whitney U test. *Indicates a p-value < 0.05, **indicates a p-value < 0.01, and ***indicates a p-value < 0.001. (**b**) Receiver operating characteristic (ROC) curve analysis and the respective areas under the curve (AUC) with 95% confidence intervals showing the performance of the RISK6 signature to discriminate between ATB patients at baseline, HD and LTBI. In the top left box, the solid and dashed lines represent the respective optimal and minimum criteria set by the WHO in the target product profile (TPP) for a screening/triage test for TB.

To investigate the use of RISK6 score as a screening and triage test for TB, we compared RISK6 scores between patients with ATB disease (n = 141), treated TB patients who have been successfully treated for TB (TREATED, n = 97, with negative sputum culture at T2 and/or T3), the individuals with LTBI (n = 26), and healthy donors (HD, n = 71). In all cohorts, RISK6 scores were significantly higher in ATB patients at baseline compared to HD (p < 0.001) and TREATED TB patients (p < 0.001) (Fig. 1a). Moreover, RISK6 score levels of TREATED patients became indistinguishable from HD. Remarkably, in the Madagascar cohort that includes the enrolled LTBI individuals, we observed a significant difference for the RISK6 scores between ATB and LTBI group (p < 0.001) but not between the LTBI group and the TREATED TB patients or the HD group. Remarkably, when we compared the RISK6 scores levels between study sites, we found that the RISK6 scores levels in ATB, TREATED TB patients and the HD recruited from Bangladesh were higher than the levels observed in the other study sites (Fig. 1a).

We then generated a receiver operating characteristic curve (ROC) and the respective areas under the curve (AUC) for each cohort to evaluate, by country, the performance of RISK6 signature as screening or triage test (Fig. 1b). First, we assessed the performance of RISK6 as a screening test for the discrimination between ATB patients and HD. Remarkably, the performance of the RISK6 signature was similar in the four different cohorts, with outstanding AUC values ranging from 90.1% (Bangladesh; 95% CI 80.7–99.4) to 96.4% (Georgia; 95% CI 90.5–100) (Fig. 1b). Secondly, ROC analysis was also performed to determine the potential of RISK6 signature as a triage test to discriminate between different stages of TB infection. Results demonstrated a powerful classifying potential to discriminate patients with ATB from LTBI or TREATED TB patients with an AUC of 92.8% (95% CI 85.6–100) and 96.1% (95% CI 91.7–100) respectively (Fig. 1b). Remarkably, we also found that the discrimination between ATB and HD was lowest in the cohort of Bangladesh when compared to other study sites (Fig. 1b).

### Performance of RISK6 signature benchmarked against the WHO TPP for a non-sputum based diagnostic test

Our findings were then benchmarked against the WHO TPP for a screening/triage test for TB that should have a minimum sensitivity of > 90% and specificity of ≥ 70%^19,27^. At a sensitivity set to > 90%, the performance of RISK6 signature as screening/triage test demonstrated specificity scores of > 70% in all cohorts, except for Bangladesh (Table 2). This shows that RISK6 signature achieves the minimal WHO TPP for non-sputum-based screening and triage tests discriminating patients with ATB from both HD and LTBI groups.

**Table 2:** Receiver operating characteristic curve analysis of the performance of the RISK6 signature to distinguish active TB cases (ATB) from healthy donors (HD) and from latent TB infected individuals (LTBI) in cohorts from Georgia, Madagascar, Lebanon, and Bangladesh.

TPP requirement		Cut-off	Sensitivity%	Specificity%	Cases, n	Controls, n	AUC	AUC 95%CI
**Screening test (ATB vs HD)**								
Georgia	Sensitivity > 90%	> 0.2583	90.6	85.7	32	7	96.4%	90.5–100%
Madagascar		> 0.3697	90.9	87	44	23	95.6%	90.9–100%
Lebanon		> 0.3171	90.5	88	21	25	94.7%	88.6–100%
Bangladesh		> 0.3625	90.9	68.8	44	16	90.1%	80.7–99.4%
All		> 0.3209	90.1	80.3	141	71	92.6%	88.8–96.3%
**Triage test (ATB vs LTBI)**								
Madagascar	Sensitivity > 90%	> 0.3697	90.9	88.5	44	26	92.8%	85.6–100%
**Initial TB diagnostic test to replace smear microscopy** (ATB (CLT^+^ AFB^−^) vs HD)								
Georgia	Sensitivity ≥ 60%	> 0.3514	63.6	100	11	7	94.8%	85.1–100%
Madagascar		> 0.4298	66.7	95.7	9	23	96.1%	90.1–100%
Lebanon		> 0.3217	75	88	4	25	90%	78.2–100%
Bangladesh		> 0.3541	75	68.8	4	16	79.7%	58.8–100%
All		> 0.3823	60.7	88.7	28	71	87.7%	80.6–94.8%
**Confirmatory test (ATB (CLT**^**+**^** AFB**^**−**^**) vs HD)**								
Georgia	Sensitivity ≥ 65%	> 0.3131	72.7	100	11	7	94.8%	85.1–100%
Madagascar		> 0.4298	66.7	95.7	9	23	96.1%	90.1–100%
Lebanon		> 0.3217	75	88	4	25	90%	78.2–100%
Bangladesh		> 0.3541	75	68.8	4	16	79.7%	58.8–100%
All		> 0.3674	67.9	87.3	28	71	87.8%	80.6–94.8%

The performance of the signature is benchmarked against the WHO TPP for a non-sputum based screening/triage test (at a sensitivity of > 90%, the minimum specificity as set out in this TPP should be ≥ 70%), for an initial TB diagnostic test to replace sputum smear (at minimum 60% sensitivity, the minimum specificity as set out in this TPP should be > 98%) and for a confirmatory test (at minimum 65% sensitivity, the minimum specificity as set out in this TPP should be > 98%)^19^.

*ATB* active TB, *LTBI* latent TB infection (were only recruited from Madagascar), *HD* healthy donors, *CLT*^*+*^ positive sputum culture, *AFB*^*−*^ negative AFB smear microscopy, *AUC* area under the curve, *CI* confidence interval, *Vs* versus.

### Performance of RISK6 as a confirmatory test for pulmonary TB disease

Our next aim was to evaluate the performance of RISK6 signature in sputum smear-negative and culture-confirmed TB individuals. Based on the TPP criteria set by the WHO as a reference^19,27^, we found that RISK6 achieved the minimal sensitivity of > 60% with 100% specificity for an initial TB diagnostic test for sputum smear-negative TB to replace smear microscopy in the cohort from Georgia (Table 2). Similarly, in the same cohort, RISK6 signature also reached the minimum criteria of 65% sensitivity and 100% specificity for a confirmatory test. However, RISK6 signature detection failed to meet these WHO requirements in the other study sites (Table 2).

**Figure 2:**
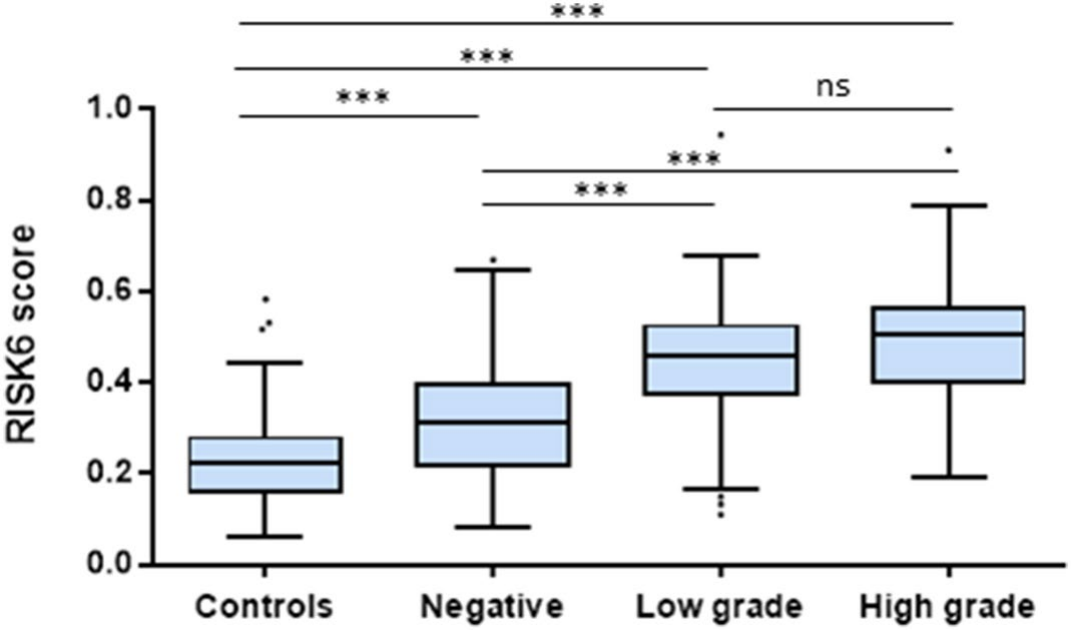
Correlation between RISK6 signature scores and mycobacterial loads determined by sputum smear microscopy in ATB patients. Boxplots comparing the RISK6 score levels stratified according to sputum smear grade: Negative smears, low grade positive smears (1+ or scanty) and high grade positive smears (2+ or 3+). Horizontal lines designate medians, boxes represent the inter-quartile ranges (IQR) and the ranges are represented by whiskers. Individual dots represent the results of patients with a RISK6 scores out of IQR. Statistical significance was calculated using Mann–Whitney U test. *Ns* non-significant, ***indicates a p-value < 0.001. *HD* Healthy donors.

As most ATB patients had a positive sputum smear microscopy with a high grade at baseline, we wondered if RISK6 scores and mycobacterial loads were correlated. We therefore performed a sub-analysis on stratified sputum smear microscopy results among ATB patients, defined as follow: negative smears, low-grade positive smears (1+ or scanty) and high-grade positive smears (2+ or 3+). RISK6 scores in the negative smear group showed a significant difference (p < 0.001) compared to HD (Fig. 2). Moreover, RISK6 scores were significantly lower (median = 0.31, IQR 0.22–0.40) in negative smears than in individuals with low- or high-grade positive smears (p < 0.001). While not statistically different (p > 0.05), RISK6 scores in the high-grade smear group were higher (median = 0.5, IQR 0.40–0.56) than in the low-grade mycobacterial load group (median = 0.46, IQR 0.38–0.52).

### Performance of RISK6 signature compared to IGRAs

Next, we assessed the performance of RISK6 signature compared to two assays based on IFN-*γ* release: the commercial QFT-P, and the non-commercial IGRAs-rmsHBHA. Compared to the QFT-P assay, the RISK6 signature achieved better performance in AUC (94.1% vs 57.2%), sensitivity (90.9% vs 50.9%) and specificity (87.8% vs 57.2%) to discriminate ATB patients from an asymptomatic population (LTBI + HD) (Table 3). However, a comparative sub-analysis indicated a lower positive (79.7%) and negative (50%) predictive values of the RISK6 signature when compared to QFT-P assay (100% and 63.9%, respectively) in detection of *Mtb-*infected individuals (ATB + LTBI) from uninfected ones (HD). Notably, the RISK6 signature showed a higher performance (AUC 90.9%, 95% CI 87.2–94.5), with 90.1% sensitivity and 72.2% specificity than the IGRAs-rmsHBHA (AUC 75.3%, 95% CI 68.6–82) that achieved lower sensitivity and specificity (83.8% and 59.8% respectively) to differentiate *Mtb*-infection status (i.e. ATB vs TREATED TB patients) (Table 3).

**Table 3:** Performance of RISK6 signature compared to Interferon-γ release assays: QuantiFERON-TB Gold Plus (QFT-P) and recombinant *Mtb*-HBHA expressed in *Mycobacterium smegmatis* (IGRAs-rmsHBHA).

Intended application	Test	Sensitivity%	Specificity%	PPV%	NPV%	Cases, n	Controls, n	AUC	AUC 95%CI
ATB vs (LTBI + HD)	RISK6	90.90	87.7	87	91.5	44	49	94.1	89.3–98.8
	QFT-P	67.50	46.9	50.9	63.9	40	49	57.2	45.2–69.1
(ATB + LTBI) vs HD	RISK6	90.00	30.4	79.7	50	66	23	77.8	68.5–87.1
	QFT-P	80.30	100	100	63.9	70	23	90.1	83.9–96.3
ATB vs treated TB	RISK6	90.1	72.2	82.5	83.3	141	97	90.9	87.2–94.5
	IGRAs-rmsHBHA	83.8	59.8	70.3	76.5	136	87	75.3	68.6–82

*ATB* active TB, *LTBI* latent TB infection, *HD* healthy donors, *vs* versus, *rmsHBH* recombinant *Mtb* HBHA expressed in, *PPV* positive predictive value, *NPV* negative predictive value.

### RISK6 as a biomarker for TB treatment monitoring

**Figure 3:**
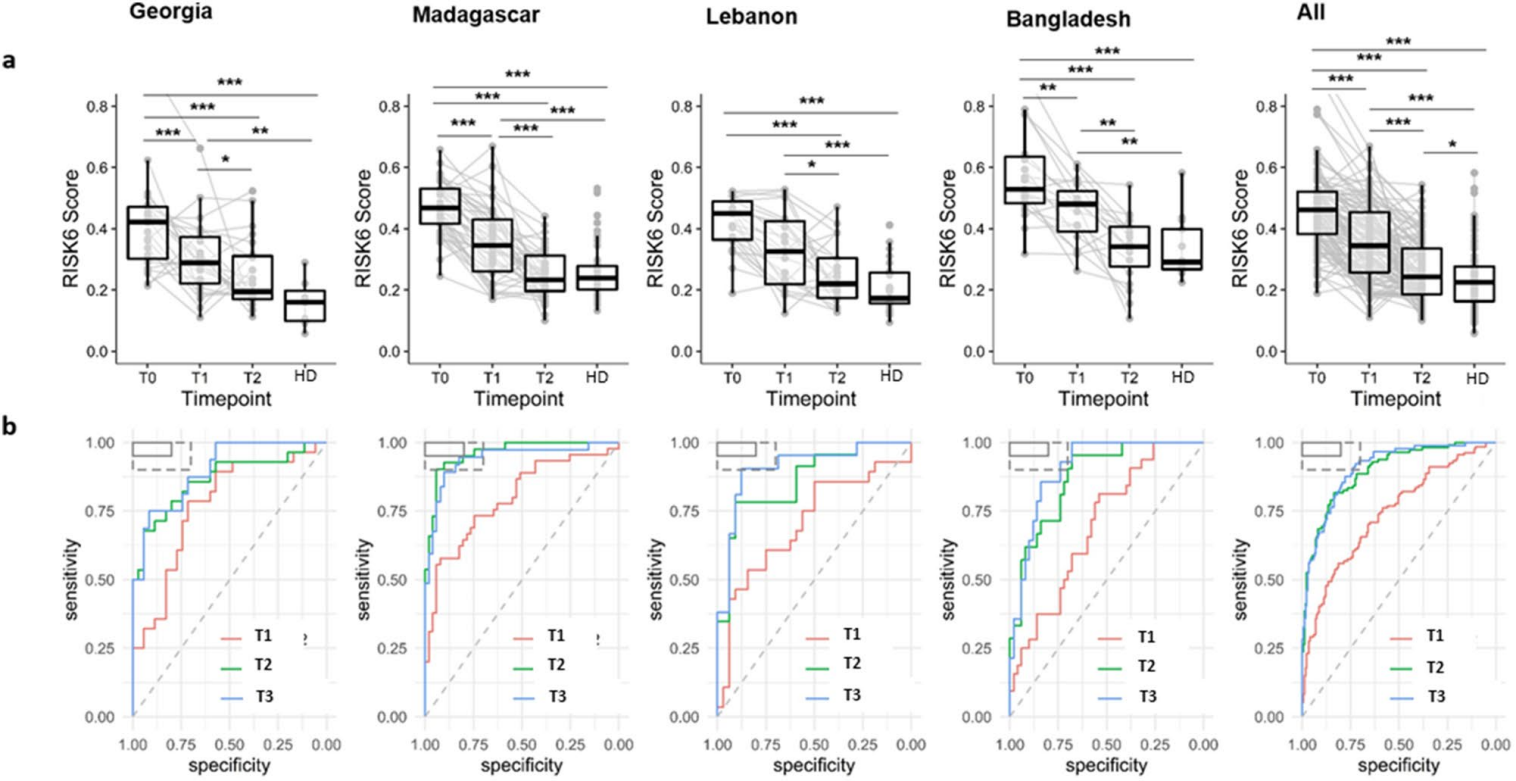
Validation of the performance of RISK6 signature as a biomarker for monitoring TB treatment response in four distinct geographical countries. (**a**) RISK6 scores were evaluated in whole blood of patients with active TB who had successfully completed their TB treatment until T2 (n = 104). Evaluation was done at baseline (T0), 2 months after treatment initiation (T1), and at the end of treatment (T2). RISK6 scores in healthy donors (HD: n = 71) were also evaluated. Horizontal lines designate medians, boxes represent the inter-quartile ranges (IQR) and the ranges are represented by whiskers. Single patient results are represented by each dot in the graph. Plotlines (grey) represent the RISK6 scores of the same patient at the three different time points. Statistical significance was calculated using Mann–Whitney U test. *Indicates a p-value < 0.05, **indicates a p-value < 0.01, and ***indicates a p-value < 0.001. (**b**) Receiver operating characteristic (ROC) curve analysis and the respective areas under the curve (AUC) with 95% confidence intervals (CI) showing the ability of the RISK6 signature to discriminate between active TB patients at baseline (T0, n = 141) and at month 2 after treatment initiation (T1, n = 117), at the end of treatment (T2, n = 104) and 2 months after treatment completion (T3, n = 79).

Patients with successful treatment (defined as negative sputum culture at T2) were selected to determine whether RISK6 signature was a clinically relevant biomarker for TB treatment monitoring. Overall, in all cohorts combined, we observed a significant drop in RISK6 scores after two months of treatment (T1, p < 0.001) and until treatment completion (T2, p < 0.001). Moreover, RISK6 scores were significantly higher in cured TB patients (T2, p > 0.05) when compared to HD, however, in each of the four cohorts, there were no significant difference between these two groups (p > 0.05) (Fig. 3a). Similarly, analytical performance demonstrated capacity of RISK6 signature to significantly discriminate patients at baseline and two months after treatment initiation (AUC 69.7%, 95% CI 57.1–79.6) (Fig. 3b and Supplementary Table 6). Noticeably, by the end of treatment, the majority of patients had lower RISK6 score levels, further enhancing the discriminatory power between ATB patients at T0 and T2 (AUC 87.1, 95% CI 77.6–94.3) and at T3 (AUC 90.4, 95% CI 82.6–96.6) (Fig. 3b and Supplementary Table 6).

Furthermore, we evaluated whether RISK6 allows the discrimination of cured TB patients (n = 104) from those with a treatment failure (defined as positive sputum culture at T2, n = 2). Thereafter, patients were stratified into drug-sensitive (DS) and drug-resistant TB (DR-TB) cases and the RISK6 signature scores were compared within these groups. We found that RISK6 scores decreased throughout treatment among DS-TB patients independently of treatment outcome (Supplementary Fig. 1). In contrast, the RISK6 score remained stable at baseline and during treatment in a DR-TB patient with a treatment failure. Importantly, RISK6 score levels during TB treatment seem to be higher in patients with treatment failure among both DS and DR-TB cases. However, in a univariate or multivariate analyses, no significant association of the RISK6 score at baseline with treatment failure was found (Supplementary Table 8).

## Discussion

TB remains one of the major infectious causes of death globally. In this study, we aimed to evaluate the relevance of RISK6, a PCR-based six-gene blood transcriptomic signature^26^, in the context of TB diagnosis and treatment monitoring. This was conducted in four independent cohorts enrolling ethnically and geographically diverse participants, including ATB patients, LTBI individuals, and HD, in both high- and low-TB incidence settings.

We first evaluated the performance of RISK6 signature as a screening test for TB and showed that it displayed similar performance in the four different cohorts with excellent near-identical ROC AUC values (> 90.1%). Furthermore, RISK6 signature satisfied the minimum criteria set by the WHO TPP for a non–sputum-based screening test^19^. Notably, our findings suggest that, compared to IGRAs, the RISK6 signature showed a better performance as a screening test for discriminating between ATB patients and HD. Importantly, compared to previous RISK6 results reported by Penn-Nicholson et al*.*^26^, our study found similar data in terms of score range and score changes over time despite the heterogeneity of both cohorts and study designs. In addition, marked technical differences are also apparent between our studies: we performed the RISK6 scores measurements on RNA manually isolated from whole blood collected directly in Tempus Blood RNA tubes and from blood samples first collected in lithium heparin tube and then transferred in Tempus Blood RNA Tubes, while this measurement was done by Penn-Nicholson et al*.* using RNA extracted manually or by an automated processes from whole blood collected in PAXgene Blood RNA tubes. Collectively, these results highlight the robustness of this PCR-based host-blood transcriptomic signature.

Besides, the higher RISK6 score levels detected in the cohort of Bangladesh compared to the other study sites was a remarkable result. We hypothesized that these RISK6 scores observed in Bangladesh may be influenced by the differing epidemiology, geographical locations as well as differences in gene expression levels between ethnic populations that may have contributed to a stronger transcriptomic signal in Bangladesh.

Our AUC data showed that RISK6 scores had a powerful ability to distinguish ATB from HD, with better or equal results to what was found with other transcriptomic signatures^28–34^. Moreover, while these previous signatures have shown promise as diagnostic tests, it should be noted that results of a three gene signature were not generalizable^28,34^, while other signatures^33^ require measurement of a high number of genes, thus limiting their possible application in resource-limited settings. Moreover, while RISK6 signature seems to meet or exceeded the TPP criteria based on each of our four cohorts, only two among the previous signatures (Sweeney3^28^ and Sambarey10^32^) satisfied the sensitivity and specificity TPP criteria set by the WHO for a triage test^35^. However, it would be interesting to validate those signatures in other independent cohorts^28,36^.

An important finding of our study is that RISK6 signature allowed to stratify TB patient’s stages. Thus, when applied to the cohort of Madagascar, the only one including LTBI cases, the RISK6 signature demonstrated a significantly higher score in ATB individuals at baseline compared to those with LTBI. This is consistent with a previous study showing that a 3-gene transcriptomic signature was significantly higher in ATB patients versus LTBI^28^ individuals, in addition to a 20-gene signature set that also discriminated ATB patients from LTBI and healthy controls^18^. In the same way, some gene-signatures were also evaluated^18^ and showed high specificity and sensitivity to distinguish ATB patients from those with LTBI^23,28,31,33^. In our study, at > 90% sensitivity, RISK6 signature discriminated ATB from both LTBI and HD with a specificity > 70% which met the WHO TPP for a triage test for TB. Besides, no significant differences in the classification performance of RISK6 signature were observed between LTBI and HD, in line with recent transcriptomic studies demonstrating failure in discriminating LTBI from HD^18,28^. Moreover, while no previous studies has compared the levels of a transcriptomic signature between LTBI and treated TB patients, our data showed that the RISK6 signature reached the same score levels in treated TB patients when compared to LTBI individuals. Hence, it will be of interest to validate RISK6 signature in cohorts with larger number of latently infected individuals.

An additional finding of our study is that RISK6 signature also achieved the minimal WHO criteria in the Georgia cohort, for (i) an initial TB diagnostic test for sputum smear-negative TB to replace smear microscopy, using culture-confirmed TB as a gold standard (ii) and a non-sputum-based confirmatory test for sputum smear-negative TB. In this context, Turner et al.^37^ reported a comparison of 27 signatures in cohorts of 181 patients for discriminating TB and no TB disease. They found that no previously published signatures achieved the minimal WHO sensitivity (65%) and specificity (98%) performance for a non-sputum-based confirmatory test for sputum smear-negative TB. Thus, our results are promising but further validation of RISK6 signature in larger cohorts will allow testing such performance. Furthermore, we found that ATB patients with low- or high-grade positive smears had significantly higher RISK6 scores compared with those with negative smears. Similarly to previous reported results with either Xpert MTB/RIF test or the C-reactive protein (CRP) concentration measurements^38,39^, our findings suggest that RISK6 signature scores directly correlate with sputum smear grade, and may possibly represent a useful tool in the identification of patients with high transmission risk.

In the present study, we also attempted to compare the performance of different TB blood-based tests; RISK6 versus two IGRAs (QFT-P and IGRAs-rmsHBHA). Our results indicate that the performance of RISK6 was greater than that of QFT-P assay for ATB case-finding. Given that QFT-P was not recommended for the diagnosis of ATB but for LTBI diagnosis, we and others have shown that this assay is a better indicator for the detection of *Mtb* infection^12,40,41^.

Our next aim was to evaluate variations in the RISK6 scores throughout successful treatment. We found that the RISK6 signature scores were significantly higher in ATB at baseline compared to HD, and continued to decrease progressively until the end of treatment reaching scores obtained in HD. Moreover, we also demonstrated that the RISK6 signature enables discrimination with high accuracy between untreated (T0), treated (T1 and T2), and post-treated (T3) TB patients who achieved a clinical cure. Taken together, these results showed the RISK6 genes might be modulated during anti-TB treatment as early as 2 months. Notably, the well-established data by Penn Nicholson et al*.*^26^ also included additional earlier time points (week 1 and week 4) and found that RISK6 signature scores decrease over the course of successful treatment as early as 1 week. Data obtained with RISK6 is consistent with previous studies showing that transcriptomic signatures could be used as a powerful tool to monitor TB treatment response^30,42–46^. In this context, it has been previously reported that reduced gene expression levels occurred rapidly during the first and the second weeks of TB treatment^47,48^. An additional report showed that ATB gene set decreased after 4 months of anti-TB treatment, however, no tests were performed at earlier time points, or during TB treatment course^49^. To note, we showed that the RISK6 signature scores returned to normal levels (compared to HD) after 6 months of treatment, which confirmed previous data^26^ but contrasted with another transcriptomic study showing that normal levels were reached 12 months after the treatment initiation^30^. Subsequently, these results indicate that RISK6 scores significantly stratified end of treatment from pre-treatment baseline. Taken together, our findings suggest that RISK6 signature could be used as a useful tool to monitor the response to anti-TB treatment. It may represent a potential alternative of the current tests used to assess TB treatment efficacy and used comparing its result with those obtained by sputum culture that are crucial to evaluate drug resistance occurrence.

Remarkably, RISK6 relies on the use of qRT-PCR that could detect low levels of gene expression^50^ and could be integrated into clinical poor settings in contrast to other complex methods. Besides, this signature requires the measurement of a small number of genes with subsequent reduced complexity and costs. Moreover, a key advantage of RISK6 is that it is a blood-based test, which is an easily accessible sample. Blood transcriptomic tests will improve the diagnosis of TB allowing faster treatment and thus reduction of transmission, especially in children, HIV co-infected TB patients and paucibacillary pulmonary TB patients. In such populations, microbiological tests are not always feasible due to the limited ability to produce good quality sputum samples or due to low bacterial loads in their samples. In the future, it will be of interest (i) to evaluate if RISK6 is able to predict the risk of progression to TB as demonstrated by the RISK11 signature^25^ and (ii) to assess the diagnostic performance of RISK6 signature as a prototype cartridge assay as it has already been evaluated for the 3-gene signature against a microbiological reference standard^51^.

This study was subject to several limitations. Indeed, the sample size was relatively small and LTBI individuals were recruited from only one country. Hence, validation of our findings in cohorts with larger number of LTBI individuals is required to better estimate specificities and sensitivities for a triage test. Moreover, only two patients had failed treatment. Therefore, further validation is required to better understand how RISK6 signature tracks with response to treatment. Additionally, we excluded diabetic and HIV-positive patients and immunosuppressed individuals in general and our study was restricted to adults. Thus, similar validation studies are needed for children and HIV-positive patients. Moreover, in future studies, it would be relevant to evaluate the specificity of the RISK6 scores in comparison to other respiratory diseases than TB, which is considered as most difficult to distinguish with.

In conclusion, data from this study provide strong proof that RISK6 can be applied as a non-sputum-based screening and triage test that met the WHO TPP benchmarks. This host response-based gene signature may be used for stratifying patients according to their TB infection status, as well as for monitoring patients over the course of treatment. RISK6 signature is applicable using a robust and simple qRT-PCR platform which facilitates its implementation in the clinical laboratories located in resource-poor settings. Our overall findings support the efforts to incorporate RISK6 signature into a point-of-care test ensuring rapid and accurate detection of ATB cases. Indeed, such simple tests are highly needed to reduce TB spread and transmission especially in areas with high TB burden that are usually disturbed with poverty.

## Methods

### Study design and population

This evaluation of the RISK6 signature was a nested case–control multicenter prospective cohort study evaluating the prognostic value of blood-based immunological biomarkers for monitoring TB treatment outcome. It was conducted within the GABRIEL Network^52^ in four different countries including Bangladesh, Georgia, Lebanon and Madagascar.

**Figure 4:**
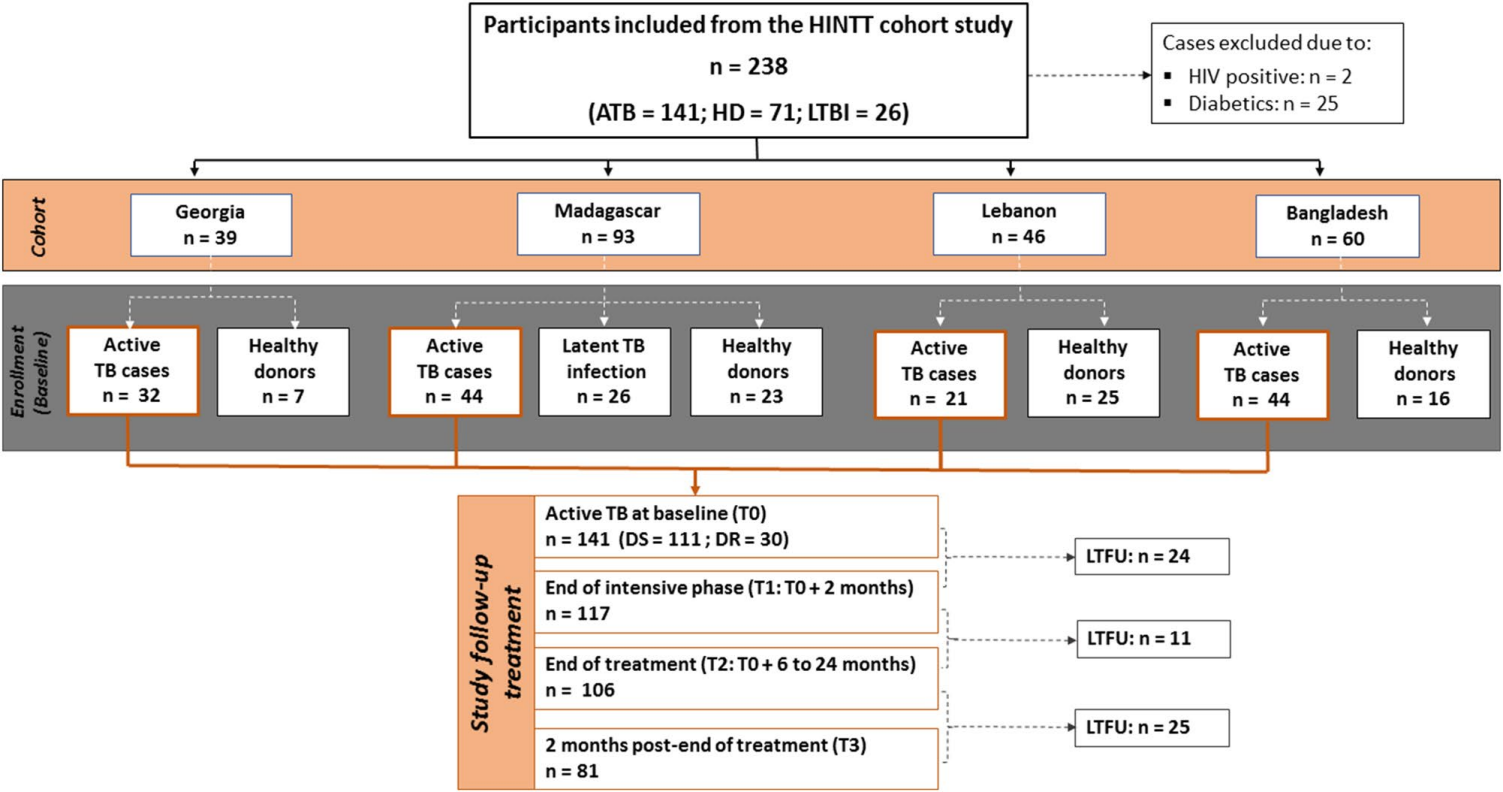
Flow diagram describing the enrollment and exclusion of participants with active TB, latent TB infection, and healthy donor participants from the different cohorts. ATB patients were followed-up at four different time points: at baseline (T0), ATB patients who didn’t their TB treatment and followed throughout antibiotic therapy: at month 2 (T1), at the end of treatment (T2), and 2 months after treatment completion (T3). *TB* Tuberculosis, *ATB* active TB, *LTBI* Latent TB infection, *HD* healthy donors, *HIV* human immunodeficiency virus, *DS* drug-susceptible, *DR* drug-resistant, *LTFU* lost to follow-up.

In total, 238 participants were recruited and followed-up between August 2018 and September 2020. Participants included patients with ATB disease (n = 141), HD (n = 71) and individuals with LTBI (n = 26). Enrolled ATB patients aged ≥ 15 years old, newly diagnosed with pulmonary ATB: scoring positive for TB following bacteriological (culture positive and/or sputum smear microscopy positive) and/or molecular analysis (GeneXpert positive results) were recruited at primary healthcare TB clinics in each country: National Center for Tuberculosis and Lung Diseases (NCTLD) in Tbilisi, Georgia; Tuberculosis screening and treatment center (CHUSSPA) related to National Tuberculosis Programs (NTPs) in Antananarivo, Madagascar; NTP centers in Tripoli and Akkar, Lebanon and International Centre for Diarrhoeal Disease Research, Bangladesh (icddr,b) in Dhaka, Bangladesh. Clinically asymptomatic healthy donors; who do not have a previous TB history and who have no recent TB contacts were also recruited in all sites. In Madagascar, participants with positive QFT-P results (IFN-γ production ≥ 0.35 IU/mL) were defined as latently *Mtb* infected individuals. Patients with negative cultures at inclusion, ATB patients with Human Immunodeficiency Virus (HIV) or with diabetes mellitus comorbidities and patients under immunocompromising treatment were excluded (Fig. 4).

Enrolled ATB patients were followed-up during the treatment course at four different time points and classified as follow: (i) ATB at baseline T0: patients who didn’t start TB treatment; (ii) treated active TB at T1 and T2: patients with ATB followed-up during the treatment and tested after 2 months of the start of the treatment (T1), and at the end of treatment (T2); (iii) treated active TB at T3: treated TB patients tested at 2 months after treatment completion.

### Ethics statement

The study protocols were reviewed and approved by the human research ethics committees in each country; Georgia, the Institutional Review Board of the National Center for Tuberculosis and Lung Diseases (NTCLD) (Reference number: IORG0009467), Madagascar, the Ministry of Public Health and the Ethical Committee for Biomedical Research (Reference number: n°099-MSANP/CERBM), Lebanon, the institutional review board of NINI hospital (Reference number: IRB-F-01) and Bangladesh, the Research Review Committee and the Ethical Review Committee of International center for diarrheal diseases and research (icddr,b). All study participants provided written informed consent. All research was performed in accordance with relevant guidelines/regulations.

### Diagnostic assessment and follow-up

ATB diagnosis was based on both bacteriological and molecular parameters. At least one sputum sample was collected at inclusion (T0) for culture testing (liquid culture media: MGIT mycobacterial growth indicator tube, BD BioSciences, NJ, USA and/or solid culture media: L–J (Lowenstein–Jensen) and also tested by microscopy for the presence of acid-fast bacilli (AFB) using the Ziehl–Neelsen staining method and/or Auramine O staining. In addition to positive culture, active TB status was defined by positive Xpert MTB/RIF (Cepheid). Patients were re-evaluated by sputum smear and culture during the intensive phase of treatment (T1) thereafter at the end of treatment (T2) and 2-months after treatment completion (T3) to confirm that they were successfully treated and cured. Drug susceptibility testing (DST) methods were performed according to standard protocols^53^.

### Demographic and clinical data collection

At enrollment and at each follow-up visit, medical history, clinical and demographic data were collected using standardized questionnaires to feed the cloud-based database system CASTOR (CASTOR Electronic Data Capture, Version 1.4, Netherlands).

### Blood collection process

A minimum of 3 mL of whole blood for transcriptomic analysis and 5 mL for the Interferon-γ release assays were drawn from each participant. For transcriptomic analysis, specimens were directly collected in Tempus Blood RNA Tubes (Applied Biosystems, 4342792), vigorously shaken, and stored at − 80 °C. Of note, in Madagascar and Bangladesh, blood samples were first collected in lithium heparin tubes and then transferred in Tempus Blood RNA Tubes for transcriptomic analysis.

### RNA extraction process and complementary DNA (cDNA) synthesis

Frozen Tempus Blood RNA tubes were thawed and RNA was manually extracted using the MagMAX™ for Stabilized Blood Tubes RNA Isolation Kit (Applied Biosystems by Thermo Fisher Scientific, 4451893) following the manufacturer’s instructions. RNA elution was performed by adding 30 µL of Elution Buffer. The purified RNA was transferred to a nuclease-free tube, assessed for quantity and quality (Nanodrop spectrophotometer), and stored at − 80 °C until needed. The cDNA was synthetized using the Applied High Capacity RNA to cDNA kit (Applied Biosystems by Thermo Fisher Scientific, 4387406). The RT reaction mix was prepared as follows: 10 µL of 2xRT buffer mix, 1 µL of 20 × RT Enzyme, and 3 µL of nuclease-free water. Then 6 µL of purified RNA/negative control samples were added and proceeded using random hexamer primers (1 h 37 °C, 5 min 95 °C and hold 4 °C). cDNA was then 1:5 diluted (nuclease-free water) and stored at − 20 °C for long-term conservation.

### Pre-amplification PCR

Prepared cDNA was pre-amplified using specific sequences of TaqMan primer-probes as previously described by Penn-Nicholson et al.^26^. 5 µL of 2 × PCR mix (TaqMan Universal PCR Master Mix 2×) (Applied Biosystems by Thermo Fisher Scientific, 4304437) with 2.5 µL of the specific primers-probes mix (PPM 0.6×), composed of primers of the 6 genes (listed in Supplementary Table 1) (Applied Biosystems by Thermo Fisher Scientific) was mixeded. Then 2.5 µL of the diluted cDNA/negative control samples were added and the mixture was incubated 10 min at 95 °C followed by 16 cycles of amplification at 95 °C for 15 s, 60 °C for 4 min, and hold at 4 °C. The pre-amplified PCR products were diluted 1:25 with nuclease-free water and stored at − 20 °C for long-term conservation.

### Quantitative Real-Time PCR (qRT-PCR) assay and gene expression analysis

For every target to amplify, 4 μL of pre-amplified DNA was subjected to a real time nucleic acid amplification using 10 µL of TaqMan Universal PCR Master Mix (Applied Biosystems by Thermo Fisher Scientific), 1 µL of primers-probe mix (20×) and 4 µL of nuclease-free water using the following conditions: 2 min at 50 °C, 10 min at 95 °C, followed by 95 °C for 15 s and 60 °C for 1 min for 40 cycles. For analytical reasons, all the PCR reactions were performed in duplicate.

### RISK6 score generation

Polymerase chain reaction signals were analyzed using CFX Manager Software version 3.1 (BioRad) in regression mode and expressed as cycle threshold (Ct) values. The step-by-step procedure for computing the 6-gene signature (RISK6) scores was performed as described by Penn-Nicholson et al*.*^26^. Briefly, the mean of Ct values was calculated for every targeted genes and combined to generate a score. The score was computed with R script available on https://bitbucket.org/satvi/risk6/src/master/.

### QuantiFERON-TB Gold Plus and IGRAs-rmsHBHA assays

1 mL of whole blood was collected directly into each of the QFT-P tubes (Qiagen, Hilden, Germany, 622526) (Nil: Negative Control, TB-Antigens (TB1/TB2) and Mitogen: Positive control) and an extra 1 mL of blood was collected in a heparin tube and stimulated with 10 µg/mL of rmsHBHA (UNICATT, Rome, Italy^10101010^). After 16–24 h incubation at 37 °C, plasma samples were harvested and stored at − 80 °C prior subjected to QFT-P ELISA (Qiagen, Hilden, Germany, 622120), following the manufacturer instructions. Briefly, 50 µL of plasma samples were tested, optical density results were compared to log-normalized values from freshly reconstituted IFN-*γ* kit standards. To account for potential immunomodulation phenomena unrelated with TB treatment, baseline IFN-*γ* level values (Nil tubes) were subtracted from antigen-stimulated IFN-*γ* values (TB1, TB2, Mitogen and rmsHBHA). According to the kit’s sensitivity range, the maximum for IFN-*γ* level values was set at 10 IU/mL and negative values were rescaled to 0.

### Statistical analysis

All statistical analyses were performed with R studio (version 4.0.3) software^54^. Graphs were created using the ggplot2 packages^55^. Statistical evaluation of the performance of RISK6 was done by calculating the Area Under the receiver operating characteristic curve (ROC AUC) and associated 95% confidence intervals (CI) using the pROC in R^56^. Discrete variables were analyzed using Fisher’s Exact test with Bonferroni’s post-hoc test. Normality was assessed using the Shapiro–Wilk Normality Test. Normal, continuous variables were analyzed with Student’s t-test. Non-normal, continuous variables were analyzed with the Mann–Whitney test or the Kruskal–Wallis rank-sum test with Dunn's Kruskal–Wallis Multiple Comparisons post-hoc test. Repeated measures of non-independent continuous variables were analyzed using the Friedman rank-sum test, with Wilcoxon–Nemenyi–McDonald–Thompson’s post-hoc test. Non-parametric data were presented as median ± IQR and the statistical significance cut-off was considered as a p value of < 0.05. For logistic regression analyses, variables were first evaluated in univariate analyses, then multivariate analyses were performed. Adjustment variables were selected as follows: sociodemographic variables of known clinical importance (e.g., sex, country of origin), TB risk factors (e.g., smoking), and additional sociodemographic variables that were at least moderately associated (p < 0.10) with the outcome in univariate analyses (e.g., prison). Irrelevant adjustment variables were then removed by backward model selection. The combination of variables that minimized the Akaike Information Criterion (AIC) for most tested predictors, while including important adjustment variables, was selected.

## Supplementary Information

Supplementary Figure 1.

Supplementary Tables.

## Data Availability

The RISK6 scores and associated clinical data for all cohorts can be found in Supplementary Tables 2–7.
