# Supplementary material for: Multi-country evaluation of RISK6, a 6-gene blood transcriptomic signature, for tuberculosis diagnosis and treatment monitoring
Source: Sci Rep. 2021 Jul 1;11:13646. doi: 10.1038/s41598-021-93059-1 (PMC8249600; doi:10.1038/s41598-021-93059-1)
Supplement: Supplementary file 1 — Supplementary Figure 1. [file 41598_2021_93059_MOESM1_ESM.docx]

**Multi-country evaluation of RISK6, a 6-gene blood transcriptomic signature, for tuberculosis diagnosis and treatment monitoring**

Rim BAYAA^1,2^*^+^, Mame Diarra Bousso NDIAYE^1,3+^, Carole CHEDID^1,4,5^, Eka KOKHREIDZE^6^, Nestani TUKVADZE^6^, Sayera BANU^7^, Mohammad Khaja Mafij UDDIN^7^, Samanta BISWAS^7^, Rumana NASRIN^7^, Paulo RANAIVOMANANA^3^, Antso Hasina RAHERINANDRASANA^8^, Julio RAKOTONIRINA^8^, Voahangy RASOLOFO^3^, Giovanni DELOGU^9^, Flavio DE MAIO^9^, Delia GOLETTI^10^, Hubert ENDTZ^11^, Florence ADER^12^, Monzer HAMZE^2^, Mohamad Bachar ISMAIL^2^, Stéphane POUZOL^1^, Niaina RAKOTOSAMIMANANA^3#^, Jonathan HOFFMANN^1#^* on behalf the HINTT working group within the GABRIEL network.

**Supplementary Figure**


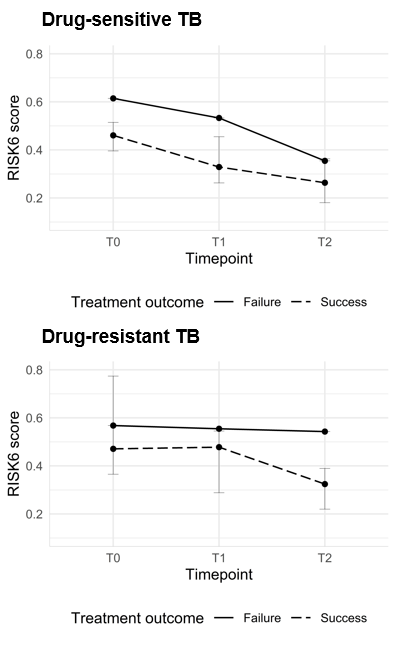


**Supplementary Figure 1: Ability of RISK6 signature to stratify TB patients according to ultimate treatment outcome.** Longitudinal Kinetics of RISK6 signature scores across TB treatment time (T0: baseline; T2: 2 months after initiation and T2: at the end of treatment) in patients with cure and those with a treatment failure, stratified into drug-sensitive (n= 95) and drug-resistant TB cases (n= 11). Dots depict medians and error bars represent the IQR.
